# HMG-COA reductase inhibitor modulates collagen GEL-contraction by hepatic myofibroblast-like stellate cell line: involvement of geranylgeranylated proteins

**DOI:** 10.1186/1476-5926-2-S1-S21

**Published:** 2004-01-14

**Authors:** Mikio Yanase, Hitoshi Ikeda, Atsushi Matsui, Eisei Noiri, Tomoaki Tomiya, Masahiro Arai, Yukiko Inoue, Kazuaki Tejima, Kayo Nagashima, Takako Nishikawa, Satoshi Kimura, Kenji Fujiwara, Marcos Rojkind, Itsuro Ogata

**Affiliations:** 1 Department of Gastroenterology, Faculty of Medicine, University of Tokyo, Tokyo 113-0033, Japan; 2 Third Department of Internal Medicine, Saitama Medical School, Saitama, Japan; 3 Department of Nephrology, Faculty of Medicine, University of Tokyo, Tokyo, Japan; 4 Department of Infection Control and Prevention, Faculty of Medicine, University of Tokyo, Tokyo, Japan; 5 Experimental Pathology Section, Department of Clinical Investigation, Walter Reed Army Medical Center, and Department of Biochemistry, Uniformed Services University of the Health Sciences, Washington, DC, USA; 6 Department of Internal Medicine, Kawakita General Hospital, Tokyo, Japan

## Introduction

Recently, cholesterol-independent effects of HMG-CoA reductase inhibitors (statins) have been clarified, such as modulation of cell morphology and/or cell-substrate attachment [1]. Some of these effects are suggested to be mediated by intermediate compounds of the cholesterol synthesis, i.e., the isoprenoids [2].

Here, we investigated the effect of HMG-CoA reductase inhibitor and/or the related compounds of the cholesterol synthesis pathway on collagen gel-contractility, cell morphology, and/or cell-substrate attachment of hepatic stellate cells (HSCs), using myofibroblast-like stellate cell line derived from a CCl_4_-induced cirrhotic rat liver.

## Methods

### Materials

Simvastatin [3] was kindly provided by Merck & Co., Inc. (Rahway, NJ) and was converted to open acid form before use. Farnesylpyrophosphate (FPP) and geranylgeranyl-pyrophosphate (GGPP) were obtained from Sigma Chemical Co. (St. Louis, MO). Prenyltransferase inhibitors; FTI-277, and GGTI-286, were from Calbiochem (La Jolla, CA). A monoclonal antibody against phosphorylated serine 19 of myosin regulatory light chain (anti-MLC-pS19) was previously described [4]. A monoclonal antibody against RhoA was from Santa Cruz Biotechnology (Santa Cruz, CA). Other materials were purchased commercially.

### Cell culture

The hepatic myofibroblast-like stellate cell line, CFSC-8B (CFSC), derived from a CCl_4_-induced cirrhotic rat liver [5], was maintained in DMEM and was supplemented with 10% fatal calf serum (FCS).

### Collagen gel-contraction assay

Contractility of CFSCs was evaluated using collagen gel lattices on 24-well culture plates as described previously [4].

### F-actin staining

For visualization of F-actin, the cells were stained overnight with TRITC-labeled phalloidin (Sigma), and then they were observed by fluorescence microscopy.

### Cell adhesion assays

Cell adhesion was measured using the electric cell-substrate impedance sensor system (ECIS; Applied BioPhysics, Inc., Troy, NY) as described previously [4].

### Immunoblotting

Cellular proteins were separated by SDS-PAGE, and immunoblotted using anti-MLC-pS19 or anti-RhoA. Immunoreactive proteins were visualized using a chemiluminescence kit (Amersham).

### Statistical analysis

Data were given as the mean value with the standard error of it, and were analyzed by the paired Student's t test.

## Results and Discussion

**Figure 1:**
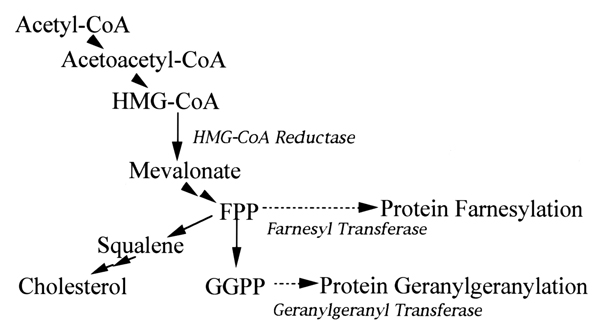
The cholesterol synthetic pathway.

HMG-CoA reductase inhibitors are widely used in patients with liver disease, such as fatty liver and/or primary liver cirrhosis, however, the effects and the mechanisms of these inhibitors on the liver remain uncertain as well in HSCs [6,7]. They block the conversion of HMG-CoA to mevalonate, the rate-limiting step in the synthesis of cholesterol, moreover, several recent studies have been focused on their cholesterol-independent effects. By modulating the initial part of the cholesterol synthesis pathway, they decrease the level of numerous important intermediate compounds including isoprenoids including FPP and GGPP (Figure 1). Isoprenoids are lipid attachments involved in post-translational modification of some proteins such as gamma-subunit of the heterotrimeric G proteins, the small G proteins as Ras, Rho, Rap, Rab, or Ral [2]. Thus, they can modulate various biological or physiological mechanisms.

In the present study we found that the addition of 10^-5^M of simvastatin attenuated the contractile activity of collagen-gel by CFSCs, which was recovered by co-addition of 10^-3^M of mevalonate, the direct metabolite of HMG-CoA. The inhibitory effect of simvastatin was also cancelled by co-addition of 10^-5^M of GGPP, but not by 10^-5^M of FPP or squalene, the late step product of the cholesterol synthesis. Moreover, the inhibitory effect was partially reproduced by addition of 10^-5^M of GGTI, not by 10^-5^M of FTI. Next, we found that cell morphology and/or the formation of stress fibers of CFSCs by F-actin staining were abrogated by simvastatin, which were maintained in the presence of mevalonate, and GGPP, but not of FPP. They were also attenuated in the presence of GGTI. We revealed further that the adhesive area of CFSCs to extracellular substrate by ECIS were reduced by simvastatin and GGTI, which were maintained in the presence of mevalonate, and GGPP. The above observations may suggest that HMG-CoA inhibitor modulates the morphological and cytoskeletal changes through the dynamic reorganization of actin filaments, and the cell-extracellular matrix interaction, resulting in the attenuation of the contraction of collagen gel lattices, and that protein geranylgeranylation is involved in this mechanism. Protein prenylation of RhoA is required to functional activities of RhoA [2]. Western blotting analyses showed that phosphorylated myosin regulatory light chain and prenylated RhoA were maintained in the presence of mevalonate, and GGPP, which were attenuated in the presence of simvastatin and/or GGTI. It may be suggested that prenylated RhoA might be associated with collagen-gel contractility, cell morphology, and/or cell-substrate attachment of CFSCs exerted by simvastatin and/or the isoprenoids.

In conclusion, HMG-CoA reductase inhibitor may modulate CFSC morphology, its attachment to surrounding extracellular matrix and its contraction by a mechanism involving protein geranylgeranylation.
